# Robotic Surgery in Urology: History from PROBOT^®^ to HUGO^TM^

**DOI:** 10.3390/s23167104

**Published:** 2023-08-11

**Authors:** Aldo Brassetti, Alberto Ragusa, Francesco Tedesco, Francesco Prata, Loris Cacciatore, Andrea Iannuzzi, Alfredo Maria Bove, Umberto Anceschi, Flavia Proietti, Simone D’Annunzio, Rocco Simone Flammia, Giuseppe Chiacchio, Mariaconsiglia Ferriero, Salvatore Guaglianone, Riccardo Mastroianni, Leonardo Misuraca, Gabriele Tuderti, Giuseppe Simone

**Affiliations:** Department of Urology, IRCCS “Regina Elena” National Cancer Institute, 00128 Rome, Italy; aldo.brassetti@ifo.it (A.B.); francesco.tedesco@unicampus.it (F.T.); francesco.prata@gmail.com (F.P.); loris.cacciatore@unicampus.it (L.C.); andrea.iannuzzi@unicampus.it (A.I.); alfredo.bove@ifo.it (A.M.B.); umberto.anceschi@ifo.it (U.A.); flavia.proietti@uniroma1.it (F.P.); simone.dannunzio@ifo.it (S.D.); simone.flammia@ifo.it (R.S.F.); gipeppo1@gmail.com (G.C.); mariaconsiglia.ferriero@ifo.it (M.F.); salvatore.guaglianone@ifo.it (S.G.); riccardo.mastroianni@ifo.it (R.M.); leonardo.misuraca@ifo.it (L.M.); gabriele.tuderti@ifo.it (G.T.); giuseppe.simone@ifo.it (G.S.)

**Keywords:** robotic surgery, robotic platform, urology, Da Vinci, Hugo-RAS system

## Abstract

The advent of robotic surgical systems had a significant impact on every surgical area, especially urology, gynecology, and general and cardiac surgery. The aim of this article is to delineate robotic surgery, particularly focusing on its historical background, its evolution, its present status, and its future perspectives. A comprehensive literature review was conducted upon PubMed/MEDLINE, using the keywords “robotic surgical system”, “robotic surgical device”, “robotics AND urology”. Additionally, the retrieved articles’ reference lists were investigated. Analysis concentrated on urological surgical systems for laparoscopic surgery that have been given regulatory approval for use on humans. From the late 1980s, before *daVinci*^®^ Era in 2000s, ancestor platform as Probot^®^ and PUMA 560 were described to outline historical perspective. Thus, new robotic competitors of Intuitive Surgical such as *Senhance^®^*, *Revo-I^®^*, *Versius^®^*, *Avatera^®^*, *Hinotori^®^*, and *Hugo^TM^ RAS* were illustrated. Although *daVinci*^®^ had high level competitiveness, and for many years represented the most plausible option for robotic procedures, several modern platforms are emerging in the surgical market. Growing competition through unique features of the new robotic technologies might extend applications fields, improve diffusion, and increase cost-effectiveness procedures. More experiences are needed to identify the role of these new advancements in surgical branches and in healthcare systems.

## 1. Introduction

Even though the term “robot” may seem like a modern idea, the concept of machines operating independently has been around for centuries. Already, by 3000 years BC, the ancient Egyptians had developed water clocks that featured miniature human figures to strike bells at regular intervals. Around 400 BC, Archytus of Taremtum, the brilliant mind behind the creation of pulleys and screws, designed a wooden pigeon with the astonishing ability to fly. Moving forward to the twelfth century AD, Ismail al-Jazari, hailed as the “father of robotics” and modern-day engineering, authored *The Book of Knowledge of Ingenious Mechanical Devices* where he detailed 50 machines, along with instructions on how to construct them. Subsequent centuries witnessed an exponential growth in ingenious automated machines, ushering in a relatively prosperous era of robotic inventions that reached its apex in the fifteenth century with Leonardo Da Vinci, who fascinated the world with a plethora of robotic marvels, ranging from humanlike to animal-like designs. Although many of Da Vinci’s designs were left unfinished, they were undeniably ahead of their time and have since served as a wellspring of inspiration for modern-day robots utilized in various fields such as medicine and astronomy. In fact, the word “robot” was initially coined by Joseph Capek in 1921, in his play *Rossom’s Universal Robots*, derived from the Czech “robota” meaning “forced labor.” Over time, the term has come to reflect a repetitive task performed by machines.

In the past few decades, we’ve experienced a surge in new technological advancements such as computer assistance, robotics, automation, and virtual reality, with healthcare being the latest industry to reap the benefits. Among these advancements, the robotic platform applied to surgery is one of the most impressive, having been used in the medical field for over 30 years now, steadily becoming a new standard of care, offering positive results [1].

This article aims to outline robotic surgery’s historical background, its evolution, its present status, as well as its future perspectives.

## 2. Materials and Methods

We conducted a PubMed/MEDLINE search using the keywords “robotic surgical system”, “robotic surgical device”, “robotics AND urology” to perform a comprehensive but not systematic literature review. We also examined the reference lists of the retrieved articles. Our review focused on surgical systems for laparoscopic surgery that have obtained regulatory approval for human use and are applicable in the field of urology.

## 3. Discussion

### 3.1. Historical Background

The incorporation of robotics in surgical procedures emerged as a response to the urgency to attain telepresence and execute repetitive and precise tasks. The first need was fulfilled in 1951, through the work of Raymond Goertz at the Atomic Energy Commission (USA), who designed a remotely operated mechanical arm to handle hazardous radioactive components [2,3,4]. The second goal was attained a decade later, when Joseph Engelberger and George Devol produced for General Motors the very first Unimate^®^ industrial robot [2] These significant accomplishments were instrumental in paving the way for the integration of robotics in various other areas of industry across the globe.

The concept of utilizing robotics in surgical procedures originated more than 50 years ago, but it was not until the late 1980s that actual use began. Kwoh et al. are often credited with the honor of the first modern robotic procedure, as they utilized the PUMA 560 robotic system for neurosurgical biopsies, effectively performing stereo-tactic brain surgery [5]. Davies et al. later used the same system for the transurethral resection of the prostate, which led to the development of the PROBOT^®^. The latter was specifically conceived by Surgical Supplies Ltd. (Dairy Flat, New Zealand) to guide the motion of a rotating blade within a predefined virtual reconstruction of the prostate, obtained through ultrasound scans [6]. Though, due to the need for manual coagulation of the prostatic fossa at the end of the procedure and because of the poor accuracy of three-dimensional reconstructions of the enlarged gland, the diffusion of PROBOT^®^ was limited [5,7,8]. However, this did not prevent the application of the same technology to orthopedic prosthetic surgery, leading to the creation of ROBODOC^®^, the first robot approved by the Food and Drugs Administration (FDA) [9].

Although there was an initial interest in active robotic systems that can autonomously perform predefined tasks, the predominant type that has gained widespread use is the master–slave one, which solely relies on the surgeon’s actions, without any pre-programmed or self-governing elements [10,11,12]. This evolution can be easily understood considering the nature of surgery, which seems to be an unsuitable profession for the utilization of fully automated robotic systems. In fact, sensitivity, empathy, adaptability, and decision-making abilities displayed by doctors are indispensable qualities when operating on the delicate boundary between health and disease, between life and death. The first surgical robot of this type was conceived in the late 1980s by Dr Phil Green, of the Stanford Research Institute (SRI, later SRI International), combining technologies for three-dimensional vision (developed by the National Aeronautics and Space Administration, NASA, in the 1960s) and telepresence [13]. The first prototype (SRI Green Telepresence) consisted of two distinct segments, the telepresence surgeon’s workstation (TSW) and the remote surgical unit (RSU). The former was equipped with a stereoscopic video monitor and a pair of instrument manipulators that relayed hand movements to the RSU. The monitor itself offered a field of view of 120 degrees and required the surgeon to wear passive polarized glasses for a clear 3D image. The RSU, on the other hand, was comprised of manipulator end-effectors with interchangeable instrument tips that could be swapped out via a twist-lock system, facilitating the use of forceps, needle drivers, bowel graspers, scalpels, and cautery tips. The unit additionally featured a pair of stereographic video cameras designed to follow the surgeon’s natural line of sight. In the early 1990s, these prototypes came under the control of the Advanced Biomedical Technologies program. In those years, in fact, the Defense Advanced Research Projects Agency (DARPA) pursued the ambitious project of halving casualties on the battlefield by cutting first aid response times, without endangering the health of military doctors [14,15]. This could be achieved through the use of an armored vehicle, deployed on the front line, equipped with a RSU (MEDFAST) through which the surgeon, using a TSW installed in the second lines, could carry out “damage control surgery” interventions from the distance [16]. In June of 1993, the telepresence surgical system was presented for the first-time during field exercises at Fort Gordon in Augusta (USA). One year later, in October of 1994, the complete system was showcased at the Association of the U.S. Army Annual Convention, where attendees were encouraged to try their hand at operating on a bleeding mannequin using the SRI: remarkably, even those lacking any surgical experience were able to successfully complete a suture and knot on the tissue, highlighting the system’s inherent user-friendliness and marking the conclusion of its initial development stages [17].

The SRI system was never intended for commercialization, but rather as a research prototype. However, the extraordinary results achieved up until then did not take long to attract private investors. Thus, in the early 90s, the two companies that would dominate the scene of robotic surgery for a decade came to life.

In 1994, the Automated Endoscopic System for Optimal Positioning^®^ (AESOP), manufactured by the Computer Motion Inc. (Goleta, CA, USA), was cleared by the FDA to assist surgeons performing minimally invasive surgery. This robotic arm was specifically designed to provide direct control over the laparoscopic camera, using either a foot pedal or voice commands. It ensured a steady view of the operative field and eliminated the need for a surgical assistant, reducing the risk of fatigue during lengthy procedures [18]. Numerous reports describe its utilization in laparoscopic cholecystectomies, hernioplasties, fundoplications, and colectomies [19].

In 1995, Intuitive Surgical was founded in California (USA) by Frederick H. Moll and Robert Younge. They reworked the SRI Green Telepresence and created their first prototype, named *Lenny*, which featured three separate robotic arms attached to the operating table: two were equipped with surgical instruments, while the third arm held the camera. In 1997, *Mona*, Intuitive’s second-generation robot, became the first surgical platform employed in human trials as J. Himpens and G. Cardiere, bariatric surgeons from Saint-Blasium General Hospital in Belgium, used it to perform a cholecystectomy [20]. In 1998, a third generation of robots was introduced: the articulated wrists of the *daVinci*^®^ robotic arms, characterized by 6 degrees of freedom, enabled cardiac surgeons from the Leipzig Heart Center (Leipzig, Germany) to perform minimally invasive cardiac valve repairs and coronary artery bypass graft surgeries [21]. The introduction of EndoWrist^®^ technology has indeed marked a significant turning point in the adoption of surgical robots. In fact, conventional laparoscopy, which had already demonstrated the benefits of a minimally invasive approach since the 1980s, had one inherent major flaw: the lack of articulating instruments. This limitation made tasks like intracorporeal suturing extremely challenging. As a result, laparoscopic training required long learning curves and led to the concentration of the widest range of surgical cases in the hands of a few experienced surgeons. The advent of robotic platforms has democratized minimally invasive surgery, enabling a larger number of doctors to approach this type of procedure.

Computer Motion replied, launching the ZEUS^®^ Robotic Surgical System (ZRSS) on the market, which was obtained coupling the AESOP with two other robotic arms with four degrees of freedom. Such a “patient-side” system, affixed to the operating table, was operated through a “surgeon-side” console, capable of minimizing the resting tremor and downscaling the hand movements to a range of 2:1 to 10:1 [22]. Although the system was initially employed in a fallopian tube anastomosis, in 1998 [23], its primary focus was on cardiac surgery, including mammary artery harvest and coronary artery bypass [20,24,25,26]. On 3 September 2001, ZEUS^®^ made history by allowing the first transatlantic telesurgery: in this groundbreaking event, Jacques Marescaux, from New York (USA), successfully performed a laparoscopic cholecystectomy in Strasbourg (France) [22,27].

In 2000, the *daVinci*^®^ received FDA approval for general laparoscopic procedures, becoming the first surgical robot used in operations in the United States. The Vattikuti Institute of Detroit documented the Vattikuti Institute prostatectomy, which would later become known as the robotic-assisted prostatectomy, with positive outcomes [28,29,30,31,32]. In comparison to the ZEUS^®^ device, the *daVinci*^®^ offered a stand-alone cart housing patient-side components, stereoscopic viewer improvements, and a more ergonomic design.

**Figure 1:**
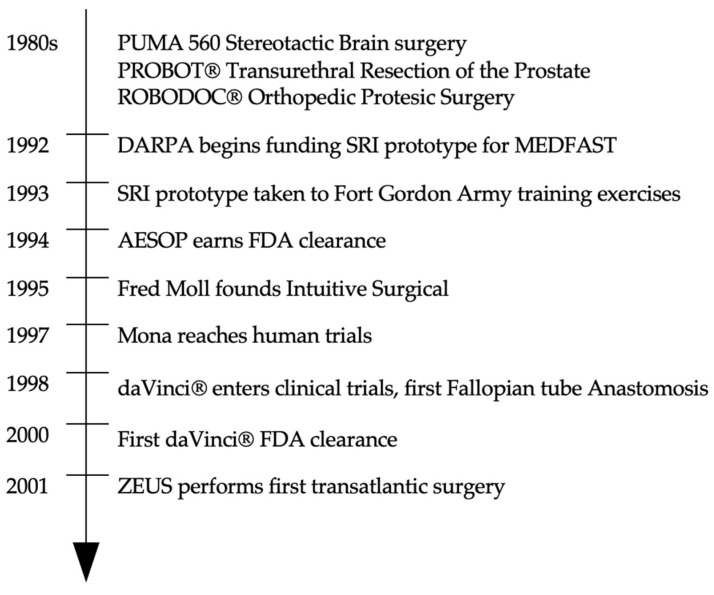
Timeline of surgical robotics development.

Intuitive and Computer Motion engaged in a legal battle for 3 years until their merger in 2003, ultimately resulting in the phasing out of ZEUS^®^ and the integration of some of its elements into later iterations of the *daVinci*^®^. A timeline of surgical robotics development is represented in Figure 1.

### 3.2. The daVinci^®^ Era

The *daVinci*^®^ robotic surgical system represented an improvement over the previous platforms. It consisted of three components: a patient cart, a surgeon console, and an image system. The system’s robotic arms were connected to the patient cart, eliminating the need to attach them to the operating table. With seven degrees of freedom and two degrees of axial rotation, the surgical instruments mimed the movement of a human wrist. The system was equipped with a 3D endoscope that captured images of the surgical field: these were projected onto the synchronized screens of the stereoscopic viewer, integrated in the surgeon console, creating a truly three-dimensional visualization without the need for specific goggles. The *daVinci*^®^ robot that received FDA approval in 2000 featured three arms [33]; a four-arm version was approved in 2002 for better control and exposure of anatomical structures, reducing reliance on a surgical assistant. At the console, two handles controlled by the surgeon were precisely connected to the arms, transmitting the surgeon’s movements to the robotic arms. Hand tremors were eliminated, and the capability to scale-down movements from 1:1 to 5:1 allowed for delicate maneuvers as required by the surgeon. The console also had a pedal unit at the bottom to accommodate different energy uses, such as monopolar or bipolar.

**Figure 2:**
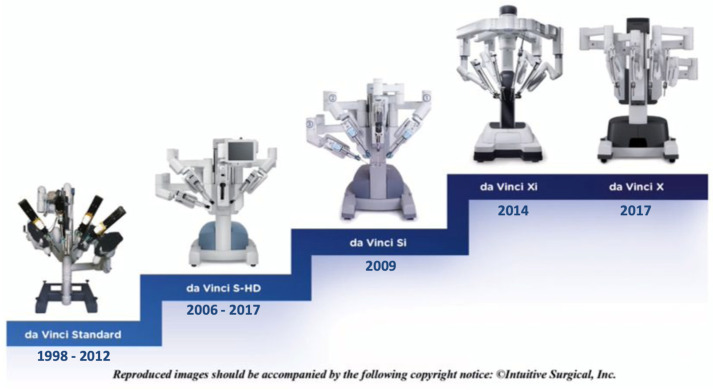
*daVinci^®^* platforms upgrading through the years, from 1998 with the first standard model until the last *X^®^* and *Xi^®^*. The new *daVinci Single–Port^®^* delineates the future of Intuitive Surgical.

The platform was upgraded over the years (Figure 2), with the da *daVinci*^®^ *S*^®^ model (2006) offering a 3D high-definition camera vision and a simplified setup, complete with an interactive touch screen display. Three years later, the *Si*^®^ model was released, introducing dual console surgery. Additionally, imaging improved substantially, with the adoption of the *Firefly*^®^ technology which allowed real-time fluorescence imaging that improved real-time decision-making during surgery, providing vital information on tissue perfusion [34]. Further platform improvements, in 2011, brought novel curved instruments, specifically designed to perform single-site surgery [35,36,37].

The most advanced system created by Intuitive Surgical, was released in 2014. The *Xi*^®^ model features a newly designed patient cart that prioritizes maximum mobility and flexibility during surgery. Its boom-mounted architecture allows for docking from any angle and improves access at any quadrant around the patient. The redesigned arms offer a broader internal range of motion, improved patient access, and minimized external collisions. Unlike earlier *daVinci*^®^ robots, the *Xi*^®^ features compact flex joints, leaving only one-fist-width spacing between each arm. This spacing can be further optimized by adjusting the patient clearance joints of each robot arm. The fourth-generation robot from Intuitive Surgical also introduced a major upgrade in visualization technology, providing a stable, immersive, highly magnified 3D-HD view of the surgical field. Surgeons have autonomous and independent control of an 8 mm endoscope, which offers a clearer view with a brighter image, higher resolution, and longer scope compared to earlier systems. The 30° endoscope can be inverted from the surgeon console without assistance, eliminating the need for removal and reinstallation. With four independent and identical robotic arms, the *Xi*^®^ system enables versatile repositioning of instruments and the endoscope at any time or port, if needed. Consolidation of this platform through the years permitted multiple applications in several unexplored fields [38].

Intuitive Surgical has recently unveiled its latest robotic model, known as the Single-Port (*SP*^®^) platform. Back in 2018, the FDA granted approval for the SP system to be utilized in urology patients. Subsequently, numerous case reports have documented the remarkable achievements of this system in tackling intricate urological procedures such as prostatectomy, donor nephrectomy, and cystectomy [37,39,40,41,42,43,44,45].

### 3.3. The New Robots

In recent years, a handful of companies have made attempts to create robotic systems (Table 1) that could potentially rival the dominance of the *daVinci*^®^, although they have not yet reached a level of competitiveness.

#### 3.3.1. Senhance^®^

The initial development of the Senhance^®^ surgical system (TransEnterix Surgical Inc., Morrisville, NC, USA) was undertaken by an Italian company (Sofar, Milan, Italy) and received the CE Mark certification in 2016 for a wide range of abdominal and noncardiac thoracic procedures. In October 2017, Senhance^®^ achieved the distinction of being the first new robotic system to gain FDA clearance since 2000 (though solely limited to general surgery and gynecology procedures). Although scientific studies comparing them are currently lacking, the Senhance^®^ robotic platform may potentially offer several advantages over the current market leader. Firstly, it utilizes a multiport configuration that can accommodate up to four independent robotic arms within separate carts. This eliminates the need for wide dedicated operating theaters and makes the system compatible with most existing ones. Additionally, the surgeon is positioned in an ergonomically designed open console with a monitor providing three-dimensional high-definition visualization though polarized glasses: this cockpit provides an unobstructed view and allows for easy interaction with the table-side assistant. Furthermore, the camera manipulation is made easier through an infrared eye-tracking system known as eye-sensing control technology, which responds to the surgeon’s eye movements, eliminating the need for dedicated controls. Moreover, the use of standard laparoscopic trocars for introducing robotic instruments enables a quick conversion to conventional laparoscopy in emergency situations. Interestingly, the system autonomously calculates the force exerted by the robotic arms on the fulcrum of the trocars, preventing excessive traction on the insertion points. Perhaps, the most significant advantage of Senhance^®^ is the availability of haptic feedback, which facilitates intracorporeal suturing and is crucial for delicate tissue handling.

In the early stages, the primary clinical applications for this system was general surgery and gynecology [46,47,48,49]. More recently, there have been reports of the successful utilization of this platform in radical prostatectomy and various other urological procedures within Europe [50,51].

#### 3.3.2. Revo-I^®^

The surgical platform known as the Revo-I^®^, developed by Meere Company Inc. in Yongin, Korea, was granted approval for human use by the Korean Ministry of Food and Drug Safety in August 2017. Similar to the *daVinci*^®^ *Si*^®^ system, this platform comprises a patient cart with four arms, a surgeon console that is enclosed, and a high-definition vision cart. The diameter of the 3D endoscope is 10 mm. The instruments, which have a diameter of 7.4 mm, offer full wrist capability and provide 7 degrees of freedom and can be reused up to 20 times [52,53]. The first study involving human subjects was published in 2018 and the Korean surgical system was used to perform a radical prostatectomy [54].

#### 3.3.3. Versius^®^

The European CE Mark was granted to the Versius^®^ surgical system (Cambridge Medical Robotics Ltd., Cambridge, UK) in March 2019. The robotic arms of this surgical system (each consisting of a shoulder, elbow, and wrist joint) are individually mounted on movable carts and remotely controlled through an open console, which allows for a 3D HD view using polarized glasses. Surgeons also receive haptic feedback from the handles. The 5 mm instruments utilized in this system offer a complete range of motion with seven degrees of freedom. Initial experiments on both cadavers and porcine models involved successfully carrying out prostate surgeries, renal surgeries, and pelvic lymph node dissections on both cadavers and porcine models [55,56,57]. The first clinical report documenting 30 cases of robotic radical hysterectomies has been recently published [58].

#### 3.3.4. Avatera^®^

Developed in Jena (Germany), the Avatera^®^ system has received clearance in Europe in November 2019, for its application in gynecology and urology minimally invasive surgeries. It consists of a patient cart which is equipped with three robotic arms for the 5 mm fully articulated disposable instruments and an additional arm for the 10 mm endoscope. All these instruments offer 7 degrees of freedom and are controlled by the surgeon via loop-like handles. The “open” console is provided with a 3D full HD vision, while maintaining visibility for improved communication with the surgical team. At present, there is a lack of published data regarding the utilization of the Avatera system [59,60].

#### 3.3.5. Hinotori^®^

Developed by Medicaroid Corporation in Kobe, Japan, Hinotori^®^ has obtained regulatory approval from the Japanese Ministry of Health, Labor, and Welfare as of August 2020. This master–slave robot comprises three main components: the surgeon cockpit, operative unit, and vision unit. Equipped with four robotic arms featuring numerous joints and capable of movement in eight axes, the operative unit boasts a semi-closed console design. Providing a 3D view of the surgical area, the console incorporates a microscope-like eyepiece. The surgeon wields control over the wristed instruments through loop-like handles. Further information and results from initial human trials for this robotic system are eagerly anticipated [61,62].

#### 3.3.6. Hugo^TM^ RAS

The Hugo^*TM*^ RAS system (Medtronic, Minneapolis, MN, USA) consists of a console with two arm-controllers that are operated with a grip similar to a pistol. It also has a footswitch that controls the camera, energy source, and reserve arm. The system includes four separate arm carts, each with six joints to increase the range of motion. Additionally, it uses specific 3D glasses for head tracking technology. The system’s first clinical case occurred in 2021 in Chile and it received approval for use in gynecological and urological procedures in the European Economic Area (EEA) in 2022, although it has not yet been approved by the FDA in the United States.

The initial series was conducted in India and reported by Ragavan and Mottrie, with a total of 7 cases performed, including radical prostatectomies, simple prostatectomy, radical nephrectomy, and simple nephrectomy [63]. A nonrandomized study comparing Radical prostatectomy outcomes between the Hugo^TM^ RAS and the Da Vinci system found no differences in total operative time or console time. The authors note that the docking process took longer with the Hugo^TM^ RAS, but the system’s independent arms provide better flexibility and more workspace for the assistant [64]. Gallioli et al. published a series of 10 patients who underwent robotic-assisted partial nephrectomy using the Hugo^TM^ system. One case required conversion to laparoscopy, and the patient later underwent selective arterial embolization due to a bleeding pseudoaneurysm. No other complications were reported [65]. While the literature is constantly growing with several case reports [66] and case series regarding this new platform, it would be interesting to effectively assess the diffusion, costs, and earnings for the hospital who bought this novel robot [67].

## 4. Conclusions

The last thirty years, since the first surgical procedure performed by robots on humans, have been crucial for the development of the master–slave robotic platform concept. Despite the undeniable technical advantages and significant clinical benefits of robotic surgery, the integration of robotics into daily clinical practice will ultimately rely on the publication of randomized clinical trials that demonstrate significant clinical advantages.

The *daVinci*^®^ system has been unrivaled in robotic surgery since it was approved in 2000. However, there is evidence of growing competition and diversity within the field. Despite its widespread success, there are alternative options available with unique features like an open console, modularity, compatibility with traditional instruments, reduced size, and reduced costs. As clinical experience advances and technology evolves, the role of these new systems in different surgical fields and healthcare systems will become clearer.

## Figures and Tables

**Table 1:** Main characteristics of the different robotic platforms currently available.

Robotic Platform	Number of Robotic Arm Carts	3D Vision	Haptic Feedback	Special Features
*Da Vinci Xi^®^*	1	Yes (HD separate screens for each surgeon eye integrated in the console)	No	The Firefly utilizes a near-infrared technology to enable the surgeon to stimulate the injected dye, causing it to emit fluorescence.
*Da Vinci Sp^®^*	1	Yes (HD separate screens for each surgeon eye integrated in the console)	No	Is currently the only robot approved for human use, specifically designed for single-port surgery.
*Senhance^®^*	Up to 4, independent	Yes (3DHD screen ad polarized goggles)	Yes	Can be docked to conventional laparoscopic trocars. Each cart can calculate the force exerted on the trocar insertion point. The “eye-sensing control” technology allows the surgeon to control the camera through the movements of his/her own eyes.
*Revo-I^®^*	1	Yes	No	Full wrist capability with 7 degrees of freedom. Can be reused up to 20 times.
*Versius^®^*	Up to 4, independent	Yes (3D HD view using polarized glasses)	Yes	Robotic arms are mounted on movable carts and remotely controlled through an open console. Complete range of motion with 7 degrees of freedom.
*Avatera^®^*	1	Yes	No	Instruments are controlled by the surgeon via loop-like handles. “Open” console with a 3D full HD vision, for a better communication with the surgical team.
*Hinotori^®^*	1	Yes	No	Robotic movements in eight axes. Semi-closed console design, incorporating a microscope-like eyepiece. Instruments are controlled by the surgeon via loop-like handles
*Hugo^TM^ RAS*	Up to 4, independent	Yes (specific 3D glasses for head tracking technology)	No	Instruments are controlled by the surgeon via a grip like a pistol. Footswitch that controls the camera, energy source, and reserve arm. Each arm carts have six joints to increase the range of motion.

## Data Availability

This article contains no data or material other than the articles used for the review and referenced.

## References

[1] Hockstein N.G., Gourin C.G., Faust R.A., Terris D.J. (2007). A History of Robots: From Science Fiction to Surgical Robotics. J. Robot. Surg..

[2] Leal Ghezzi T., Campos Corleta O. (2016). 30 Years of Robotic Surgery. World J. Surg..

[3] Goertz R.C. (1953). Remote-Control Manipulator.

[4] Goertz R.C. (1952). Fundamentals of General Purpose Remote Manipulators. Nucleonics.

[5] Kwoh Y.S., Hou J., Jonckheere E.A., Hayati S. (1988). A Robot with Improved Absolute Positioning Accuracy for CT Guided Stereotactic Brain Surgery. IEEE Trans. Biomed. Eng..

[6] Davies B.L., Hibberd R.D., Ng W.S., Timoney A.G., Wickham J.E.A. (1991). The Development of a Surgeon Robot for Prostatectomies. Proc. Inst. Mech. Eng. H.

[7] Stefano G.B. (2017). Robotic Surgery: Fast Forward to Telemedicine. Med. Sci. Monit..

[8] Harris S.J., Arambula-Cosio F., Mei Q., Hibberd R.D., Davies B.L., Wickham J.E.A., Nathan M.S., Kundu B. (1997). The Probot—An Active Robot for Prostate Resection. Proc. Inst. Mech. Eng. H.

[9] Paul H.A., Bargar W.L., Mittlestadt B., Musits B., Taylor R.H., Kazanzides P., Zuhars J., Williamson B., Hanson W. (1992). Development of a Surgical Robot for Cementless Total Hip Arthroplasty. Clin. Orthop. Relat. Res..

[10] Sackier J.M., Wang Y. (1994). Robotically Assisted Laparoscopic Surgery. From Concept to Development. Surg. Endosc..

[11] Ewing D.R., Pigazzi A., Wang Y., Ballantyne G.H. (2004). Robots in the Operating Room—The History. Semin. Laparosc. Surg..

[12] Unger S.W., Unger H.M., Bass R.T. (1994). AESOP Robotic Arm. Surg. Endosc..

[13] Parekattil S.J., Moran M.E. (2010). Robotic Instrumentation: Evolution and Microsurgical Applications. Indian. J. Urol..

[14] Green P.S., Hill J.W., Jensen J.F., Shah A. (1995). Telepresence Surgery. IEEE Eng. Med. Biol..

[15] Zajtchuk R.G.C. (1995). Part IV. Surgical Combat Casualty Care: Anesthesia and Perioperative Care of the Combat Casualty, Vol. 1. Textbook of Military Medicine.

[16] George E.I., Brand T.C., LaPorta A., Marescaux J., Satava R.M. (2018). Origins of Robotic Surgery: From Skepticism to Standard of Care. JSLS.

[17] Satava R.M. (2003). Robotic Surgery: From Past to Future—A Personal Journey. Surg. Clin. N. Am..

[18] Kavoussi L.R., Moore R.G., Adams J.B., Partin A.W. (1995). Comparison of Robotic versus Human Laparoscopic Camera Control. J. Urol..

[19] Bacá I., Schultz C., Grzybowski L., Göetzen V. (1999). Voice-Controlled Robotic Arm in Laparoscopic Surgery. Croat. Med. J..

[20] Reichenspurner H., Damiano R.J., Mack M., Boehm D.H., Gulbins H., Detter C., Meiser B., Ellgass R., Reichart B. (1999). Use of the Voice-Controlled and Computer-Assisted Surgical System ZEUS for Endoscopic Coronary Artery Bypass Grafting. J. Thorac. Cardiovasc. Surg..

[21] Morrell A.L.G., Morrell-Junior A.C., Morrell A.G., Mendes J.M.F., Tustumi F., De-Oliveira-e-silva L.G., Morrell A. (2021). The History of Robotic Surgery and Its Evolution: When Illusion Becomes Reality. Rev. Col. Bras. Cir..

[22] Marescaux J., Rubino F. (2003). The ZEUS Robotic System: Experimental and Clinical Applications. Surg. Clin. N. Am..

[23] Falcone T., Goldberg J., Garcia-Ruiz A., Margossian H., Stevens L. (1999). Full Robotic Assistance for Laparoscopic Tubal Anastomosis: A Case Report. J. Laparoendosc. Adv. Surg. Tech. A.

[24] Hashizume M., Konishi K., Tsutsumi N., Yamaguchi S., Shimabukuro R. (2002). A New Era of Robotic Surgery Assisted by a Computer-Enhanced Surgical System. Surgery.

[25] Hanly E.J., Talamini M.A. (2004). Robotic Abdominal Surgery. Am. J. Surg..

[26] Hagen M.E., Stein H., Curet M.J., Kim C.H. (2014). Introduction to the Robotic System. Robotics in General Surgery.

[27] Marescaux J., Leroy J., Gagner M., Rubino F., Mutter D., Vix M., Butner S.E., Smith M.K. (2001). Transatlantic Robot-Assisted Telesurgery. Nature.

[28] Tewari A., Menon M. (2003). Vattikuti Institute Prostatectomy: Surgical Technique and Current Results. Curr. Urol. Rep..

[29] Luciani L.G., Chiodini S., Mattevi D., Cai T., Puglisi M., Mantovani W., Malossini G. (2017). Robotic-Assisted Partial Nephrectomy Provides Better Operative Outcomes as Compared to the Laparoscopic and Open Approaches: Results from a Prospective Cohort Study. J. Robot. Surg..

[30] Hyams E.S., Mufarrij P.W., Stifelman M.D. (2008). Robotic Renal and Upper Tract Reconstruction. Curr. Opin. Urol..

[31] Cohen A.J., Pariser J.J., Anderson B.B., Pearce S.M., Gundeti M.S. (2015). The Robotic Appendicovesicostomy and Bladder Augmentation: The next Frontier in Robotics, Are We There?. Urol. Clin. N. Am..

[32] Robot Wars: $60B Intuitive Surgical Dominated Its Market for 20 Years. Now Rivals like Alphabet Are Moving in. https://www.forbes.com/sites/michelatindera/2019/02/14/intuitive-surgical-stock-robot-surgery-da-vinci-alphabet-jnj-ceo-gary-guthart/#565d4979a37b.

[33] US Food and Drug Administration (2000) 510 (k) Clearances. http://www.accessdata.fda.gov/scripts/cdrh/cfpmn/pmn.cfm?ID=K990144.

[34] Hellan M., Spinoglio G., Pigazzi A., Lagares-Garcia J.A. (2014). The Influence of Fluorescence Imaging on the Location of Bowel Transection during Robotic Left-Sided Colorectal Surgery. Surg. Endosc..

[35] Freschi C., Ferrari V., Melfi F., Ferrari M., Mosca F., Cuschieri A. (2013). Technical Review of the Da Vinci Surgical Telemanipulator. Int. J. Med. Robot..

[36] Oleynikov D. (2008). Robotic Surgery. Surg. Clin. N. Am..

[37] Gosrisirikul C., Don Chang K., Raheem A.A., Rha K.H. (2018). New Era of Robotic Surgical Systems. Asian J. Endosc. Surg..

[38] Brassetti A., Ragusa A., Bove A.M., Anceschi U., Ferriero M., Guaglianone S., Mastroianni R., Misuraca L., Tuderti G., Gallucci M. (2023). Robot-Assisted Transperitoneal Repair of a Recto-Vesical Fistula, a Case Report. Urol. Video J..

[39] LaMattina J.C., Alvarez-Casas J., Lu I., Powell J.M., Sultan S., Phelan M.W., Barth R.N. (2018). Robotic-Assisted Single-Port Donor Nephrectomy Using the Da Vinci Single-Site Platform. J. Surg. Res..

[40] Gaboardi F., Pini G., Suardi N., Montorsi F., Passaretti G., Smelzo S. (2019). Robotic Laparoendoscopic Single-Site Radical Prostatectomy (R-LESS-RP) with DaVinci Single-Site^®^ Platform. Concept and Evolution of the Technique Following an IDEAL Phase 1. J. Robot. Surg..

[41] Dobbs R.W., Halgrimson W.R., Talamini S., Vigneswaran H.T., Wilson J.O., Crivellaro S. (2020). Single-Port Robotic Surgery: The next Generation of Minimally Invasive Urology. World J. Urol..

[42] Covas Moschovas M., Bhat S., Rogers T., Onol F., Roof S., Mazzone E., Mottrie A., Patel V. (2020). Technical Modifications Necessary to Implement the Da Vinci Single-Port Robotic System. Eur. Urol..

[43] Agarwal D.K., Sharma V., Toussi A., Viers B.R., Tollefson M.K., Gettman M.T., Frank I. (2020). Initial Experience with Da Vinci Single-Port Robot-Assisted Radical Prostatectomies. Eur. Urol..

[44] Kaouk J., Garisto J., Eltemamy M., Bertolo R. (2019). Step-by-Step Technique for Single-Port Robot-Assisted Radical Cystectomy and Pelvic Lymph Nodes Dissection Using the Da Vinci^®^ SP^TM^ Surgical System. BJU Int..

[45] Zhang M., Thomas D., Salama G., Ahmed M. (2020). Single Port Robotic Radical Cystectomy with Intracorporeal Urinary Diversion: A Case Series and Review. Transl. Androl. Urol..

[46] Fanfani F., Restaino S., Rossitto C., Gueli Alletti S., Costantini B., Monterossi G., Cappuccio S., Perrone E., Scambia G. (2016). Total Laparoscopic (S-LPS) versus TELELAP ALF-X Robotic-Assisted Hysterectomy: A Case-Control Study. J. Minim. Invasive Gynecol..

[47] Spinelli A., David G., Gidaro S., Carvello M., Sacchi M., Montorsi M., Montroni I. (2017). First Experience in Colorectal Surgery with a New Robotic Platform with Haptic Feedback. Color. Dis..

[48] Rao P.P. (2018). Robotic Surgery: New Robots and Finally Some Real Competition!. World J. Urol..

[49] Bozzini G., Gidaro S., Taverna G. (2016). Robot-Assisted Laparoscopic Partial Nephrectomy with the ALF-X Robot on Pig Models. Eur. Urol..

[50] Kaštelan Ž., Knežević N., Hudolin T., Kuliš T., Penezić L., Goluža E., Gidaro S., Ćorušić A. (2019). Extraperitoneal Radical Prostatectomy with the Senhance Surgical System Robotic Platform. Croat. Med. J..

[51] Samalavicius N.E., Janusonis V., Siaulys R., Jasėnas M., Deduchovas O., Venckus R., Ezerskiene V., Paskeviciute R., Klimaviciute G. (2020). Robotic Surgery Using Senhance^®^ Robotic Platform: Single Center Experience with First 100 Cases. J. Robot. Surg..

[52] Lim J.H., Lee W.J., Park D.W., Yea H.J., Kim S.H., Kang C.M. (2017). Robotic Cholecystectomy Using Revo-i Model MSR-5000, the Newly Developed Korean Robotic Surgical System: A Preclinical Study. Surg. Endosc..

[53] Kim D.K., Park D.W., Rha K.H. (2016). Robot-Assisted Partial Nephrectomy with the REVO-I Robot Platform in Porcine Models. Eur. Urol..

[54] Chang K.D., Abdel Raheem A., Choi Y.D., Chung B.H., Rha K.H. (2018). Retzius-Sparing Robot-Assisted Radical Prostatectomy Using the Revo-i Robotic Surgical System: Surgical Technique and Results of the First Human Trial. BJU Int..

[55] Thomas B.C., Slack M., Hussain M., Barber N., Pradhan A., Dinneen E., Stewart G.D. (2021). Preclinical Evaluation of the Versius Surgical System, a New Robot-Assisted Surgical Device for Use in Minimal Access Renal and Prostate Surgery. Eur. Urol. Focus..

[56] Morton J., Hardwick R.H., Tilney H.S., Gudgeon A.M., Jah A., Stevens L., Marecik S., Slack M. (2021). Preclinical Evaluation of the Versius Surgical System, a New Robot-Assisted Surgical Device for Use in Minimal Access General and Colorectal Procedures. Surg. Endosc..

[57] Peters B.S., Armijo P.R., Krause C., Choudhury S.A., Oleynikov D. (2018). Review of Emerging Surgical Robotic Technology. Surg. Endosc..

[58] Puntambekar S.P., Goel A., Chandak S., Chitale M., Hivre M., Chahal H., Rajesh K.N., Manerikar K. (2021). Feasibility of Robotic Radical Hysterectomy (RRH) with a New Robotic System. Experience at Galaxy Care Laparoscopy Institute. J. Robot. Surg..

[59] Medicaroid’s Hinotori Surgical Robot System Approved in Japan. http://surgrob.blogspot.com/2020/08/medicaroids-hinotori-surgical-robot.html.

[60] News-Detail—Avateramedical. https://www.avatera.eu/en/company/news/detail?tx_news_pi1%5Bnews%5D=19&cHash=0b499a1adf30ef40b4d441aa562e0a7b.

[61] https://www.Medicaroid.Com/En/Product/Hinotori/.

[62] http://Surgrob.Blogspot.Com/2020/08/Medicaroids-Hinotori-Surgical-Robot.Html.

[63] Ragavan N., Bharathkumar S., Chirravur P., Sankaran S., Mottrie A. (2022). Evaluation of Hugo RAS System in Major Urologic Surgery: Our Initial Experience. J. Endourol..

[64] Ragavan N., Bharathkumar S., Chirravur P., Sankaran S. (2023). Robot-Assisted Laparoscopic Radical Prostatectomy Utilizing Hugo RAS Platform: Initial Experience. J. Endourol..

[65] Gallioli A., Uleri A., Gaya J.M., Territo A., Aumatell J., Verri P., Basile G., Fontanet S., Tedde A., Diana P. (2023). Initial Experience of Robot-Assisted Partial Nephrectomy with Hugo^TM^ RAS System: Implications for Surgical Setting. World J. Urol..

[66] Prata F., Ragusa A., Anceschi U., Civitella A., Tuzzolo P., Tedesco F., Cacciatore L., Iannuzzi A., Callè P., Raso G. (2023). Hugo RAS Robot-Assisted Partial Nephrectomy for High-Nephrometry Score Complex Renal Mass: Case Report and Surgical Technique. Videourology.

[67] Esperto F., Cacciatore L., Tedesco F., Testa A., Callè P., Ragusa A., Deanesi N., Minore A., Prata F., Brassetti A. (2023). Impact of Robotic Technologies on Prostate Cancer Patients’ Choice for Radical Treatment. J. Pers. Med..

